# Spectral Signatures of Protonated Noble Gas Clusters of Ne, Ar, Kr, and Xe: From Monomers to Trimers

**DOI:** 10.3390/molecules27103198

**Published:** 2022-05-17

**Authors:** Jake A. Tan, Jer-Lai Kuo

**Affiliations:** Institute of Atomic and Molecular Sciences, Academia Sinica, No. 1 Roosevelt Rd., Sec. 4, Taipei 10617, Taiwan

**Keywords:** noble gas chemistry, noble gas hydride ions, noble gas onium ions, infrared spectroscopy, proton-bound clusters

## Abstract

The structures and spectral features of protonated noble gas clusters are examined using a first principles approach. Protonated noble gas monomers (NgH^+^) and dimers (NgH^+^Ng) have a linear structure, while the protonated noble gas trimers (Ng_3_H^+^) can have a T-shaped or linear structure. Successive binding energies for these complexes are calculated at the CCSD(T)/CBS level of theory. Anharmonic simulations for the dimers and trimers unveil interesting spectral features. The symmetric NgH^+^Ng are charactized by a set of progression bands, which involves one quantum of the asymmetric Ng-H^+^ stretch with multiple quanta of the symmetric Ng-H^+^ stretch. Such a spectral signature is very robust and is predicted to be observed in both T-shaped and linear isomers of Ng_3_H^+^. Meanwhile, for selected asymmetric NgH^+^Ng’, a Fermi resonance interaction involving the first overtone of the proton bend with the proton stretch is predicted to occur in ArH^+^Kr and XeH^+^Kr.

## 1. Introduction

The noble gas elements have greatly impacted the development of modern chemistry for several reasons. First, it serves as a link between the highly electronegative halogens and the highly electropositive alkali metals, which in effect serves as a key in interpreting the periodic table [1]. Second, these noble gases have provided some of the early but important problems of valency. In the days of old, these gases were thought to be inert. They were though to have no ability to form chemical compounds. Such paradigm of the time has led to important concepts in chemistry such as the famous octet rule [2,3] and 18-electron rule in organometallic chemistry [4]. Third, the successful synthesis of the first noble gas containing compound XePtF_6_ by Neil Bartlett in 1962 has opened a new era of noble gas chemistry [5]. Since then, various noble gas containing compounds have been reported in the literature [6,7,8,9].

The recent detection of helonium (HeH^+^) and argonium (ArH^+^) ions in outer space has rejuvenated the interest to search for noble gas containing compounds in the extraterrestrial domain. Although HeH^+^ was speculated to exists since the 1970s, its detection in NG7027, a very young dense planetary nebula, was only reported in 2019. The helonium ion is the lightest heteroatomic ion and is postulated to be the first compound formed after the Big Bang [10]. Meanwhile, the argonium ion is the first noble gas molecular ion detected in outer space. Studies from Barlow and Schilke have shown that ArH^+^ is very prevalent in the Crab Nebula and diffuse interstellar clouds [11,12].

In the terrestrial domain, protonated Ng monomers (NgH^+^) were first observed in mass spectrometers as early as 1933 [13,14,15]. In the 60s and 70s, the NgH^+^ species has been an active subject in the field of ion-molecule reactions [16,17,18,19]. Early calculations of Rosmus and co-workers have predicted that the radiative lifetimes for the ground state vibrational levels for NgH^+^ are much shorter than their isoelectronic neutral counterparts (i.e., H_2_, HF, HCl, and HBr) [20,21,22]. The vibration-rotation spectrum for these species are expected to be strong. Such prediction has motivated experimental measurements for the vibration-rotation spectrum of these species [23,24,25,26,27,28,29]. Recently, Borocci et al. have examined the bonding nature of NgH^+^ using local electron energy density [30]. Furthermore, high-level simulations at the CCSD(T) and MRCI+Q levels have been performed to calculate the vibrational spectrum and rotational constants for NgH^+^ [31].

The earliest reported observation for protonated noble gas cluster was in 1972 when Bondybey and Pimentel had reported their studies on infrared absorption of interstitial hydrogen atom in solid argon and krypton matrix [32]. Their pioneering work had reported the first infrared spectrum for ArH^+^Ar and KrH^+^Kr in solid matrix. Based on their matrix isolation experiments in a deposit of Ar and H_2_, a strong band at 905 cm^−1^ is observed. In a deposit of Ar and D_2_, this band red shifts to 644 cm^−1^. However, spectral shifts from an ^36^Ar to ^40^Ar isotope substitution experiment have initially ruled out the existence of ArH^+^ and ArH^+^Ar as the absorbing species. As a result, these bands were initially assigned to vibrations of an interstitial hydrogen in the matrix. A year later, Milligan and Jacox investigated these interstitial hydrogens and confirmed that the 905 cm^−1^ and 644 cm^−1^ bands correspond to Ar_n_H^+^ and Ar_n_D^+^, respectively, [33]. However, they were not able to identify the cluster’s size. Eventually, several groups have studied the vibrational structure of NgH^+^Ng and NgH^+^Ng’ by means of matrix isolation experiments [34,35,36,37].

There has also been several computational studies conducted for proton-bound noble gas dimers. Early calculations have focused on their structure and characteristic fundamental bands [19,38,39,40,41,42,43,44,45,46,47,48,49]. The works of Grabowski et al. and Borocci et al. have provided valuable insights on the bonding for these complexes [50,51]. Spectral simulations which accounts for the anharmonic effects have also been available recently [52,53,54,55,56,57,58,59,60].

Recently, Duncan’s group have reported the first gas-phase spectrum for Ar_n_H^+^ (n = 3–7) [57]. The spectrum for Ar_3_H^+^ has a rich vibrational structure in the 900–2200 cm^−1^ region. A series of strong bands arising from the asymmetric proton stretch in combination with multiple quanta of the symmetric Ar-H stretch is responsible for this vibrational structure. In our previous works, we found that although a simulation of the ArH^+^Ar is sufficient to recover most of the experimental bands for Ar_3_H^+^, all of the experimental bands can only be explained by considering the T-shaped and linear isomers of Ar_3_H^+^ [57,58,59,60].

For the past few years, our group has been very active in studying the spectral features of protonated noble gas complexes [57,58,59,60]. We have found that in addition to the bright shared-proton stretch fundamental, there are other interesting features for protonated dimers and trimers. The infrared spectrum of NgH^+^Ng is characterized by a progression band [57,58]. Meanwhile for some asymmetric NgH^+^Ng’, a Fermi resonance involving the proton bend overtone and shared-proton stretch can be observed. In the present work, we review these spectral features and have extended the study by simulating protonated noble gas trimers. To put all these spectral features in context, the spectral features of protonated noble gas monomers, dimers, and trimers are presented in this work.

## 2. Computational Details

All geometry optimizations, frequency calculations, and single-point energy calculations for the potential energy surface (PES) construction were conducted at the CCSD(T) level. Dunning’s [61,62] aug-cc-pVTZ basis set were used for H, Ne, and Ar atoms, while Peterson and co-workers’s [63] aug-cc-pVTZ-PP basis set were used for Kr and Xe atoms. These aug-cc-pVTZ-PP basis sets were obtained from the Basis Set Exchange webpage [64,65,66]. The PP in these basis sets implies that a pseudopotential (PP) is included in its definition. This PP is a small-core energy-consistent relativistic pseudopotential, which is used to replace the (1s-2p) inner cores of Kr and (1s-3d) inner cores of Xe [63]. All of the above calculations were performed using Gaussian 16 Rev. A03 package [67].

Dissociation energies with respect to an elimination of a Ng from the protonated Ng complexes were calculated at the CCSD(T)/CBS//CCSD(T)/aug-cc-pVTZ level. The above notation means that we have used the minimum geometries at the CCSD(T)/aug-cc-pVTZ level and performed single point energy calculations at the aug-cc-pVQZ and aug-cc-pV5Z basis sets. Afterward, these energies were then extrapolated at the complete basis set (CBS) limit. The Hartree-Fock (HF) energies are extrapolated separately from the CCSD(T) correlation energies using the following functions.

(1) $$E_{n}^{HF}=E_{CBS}^{HF}+Ae^{-nB}$$

(2) $$E_{n}^{Corr}=E_{CBS}^{Corr}+Cn^{-3}$$

The CBS extrapolation was performed using the Molpro 2020.1 package [68,69,70,71,72].

We now describe the details of our anharmonic simulation. Normal coordinates [73,74] were used to describe the vibrational coordinates in this work. These coordinates were obtained from the frequency calculations at the equilibrium geometries. There are no kinetic energy coupling terms when normal coordinates are used, and all inter-mode couplings are included in the potential energy component. The vibrational Hamiltonian used in this work is given in Equation (3).

(3) $$\hat{H}=\frac{-\hbar^{2}}{2}\sum_{i=1}^{k}\frac{1}{\mu_{i}}\frac{\partial^{2}}{\partial Q_{i}^{2}}+\hat{V}\left(\vec{Q}\right)$$

where $\mu_{i}$ is the reduced mass of mode *i*, and $Q_{i}$ is the normal coordinate of mode *i*. We note that the vibrational Hamiltonian used in this work is a simplification of the Watson Hamiltonian [75], in which the vibrational angular momentum terms are ignored in the present treatment.

The potential energy in Equation (3) was constructed by scanning a set of Gauss–Hermite quadrature grids along the normal modes. Harmonic oscillator functions from frequency calculation were used to define these grids. The details on how this is done is written elsewhere [58,60,76]. For the NgH^+^ species, a one-dimensional potential curve was built at the CCSD(T) level. For larger clusters (i.e., NgH^+^Ng, NgH^+^Ng’, and Ng_3_H^+^), a multidimensional PES was constructed using the n-mode representation (n-MR) approach [77,78]. A four-mode representation of the potential is used. Furthermore, a multilevel scheme was used for these PES [78,79,80,81,82,83]. The more important 1-mode and 2-mode couplings were constructed at the CCSD(T) level, while the less important 3-mode and 4-mode couplings were constructed at the MP2 level. In these PES calculations, the aug-cc-pVTZ basis set was used for H, Ne, and Ar atoms, while the aug-cc-pVTZ-PP basis set was used for Kr and Xe atoms. Although a larger basis set would provide a more accurate treatment of the anharmonic frequencies, the purpose of the present work is to compare the spectral features of the protonated noble gas clusters from monomers to trimers. Basis set effects would affect the predicted peak positions of the present study, but the spectral features would be robust with respect to the basis set’s size.

The Schrödinger equation of the Hamiltonian in Equation (3) was then solved using the standard harmonic oscillator discrete variable representation (DVR) method [84,85,86,87]. The vibrational states were obtained by diagonalizing the Hamiltonian matrix in DVR basis using the ARPACK in Scipy [88,89].

Transition intensities are then estimated in terms of integrated absorption coefficient $A_{f0}$ as given in Equation (4) [90].

(4) $$A_{f0}=\left(\frac{\pi N_{A}}{3c\varepsilon_{0}\hbar^{2}}\right)\nu_{f0}{\left|\langle \psi_{0}|\left.\hat{\mu }\right|\psi_{f}\rangle \right|}^{2}.$$

where $N_{A}$ is the Avogadro’s number, *c* is the speed of light in vacuum, $\epsilon_{0}$ is the permittivity in vacuum, *ℏ* is the reduced-Planck’s constant, $\nu_{f0}$ is the transition frequency, $\hat{\mu }$ is the dipole moment operator, and $\psi_{0}$ and $\psi_{f}$ are the ground and final states involved in a transition.

## 3. Results and Discussion

### 3.1. Proton-Bound Noble Gas Monomers

**Figure 1:**
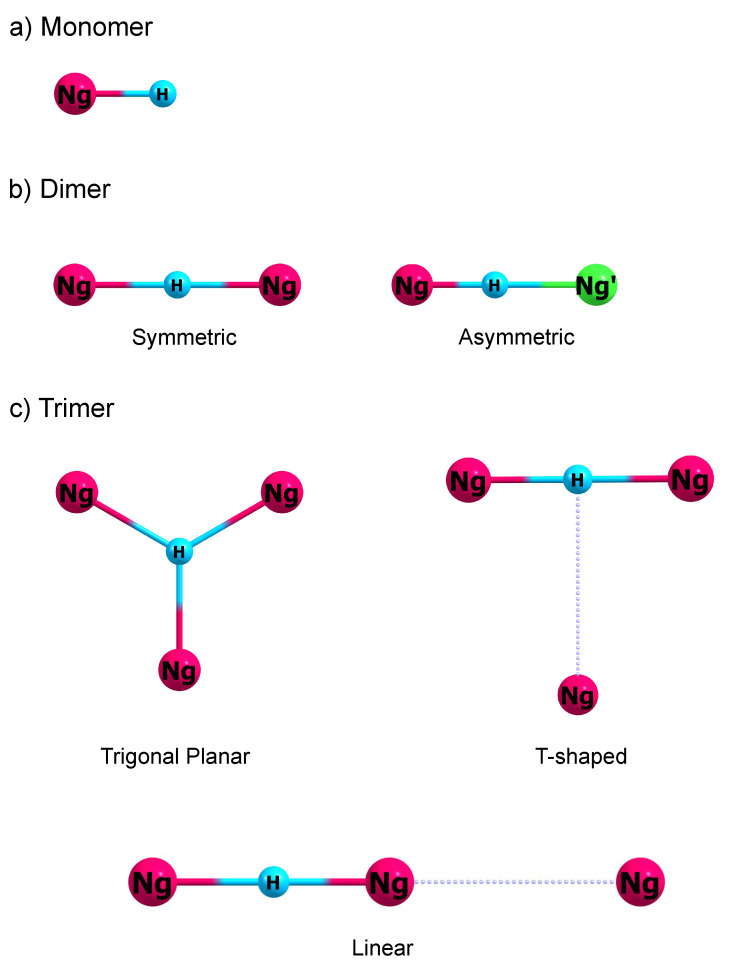
The structures of protonated noble gas clusters: (**a**) monomer, (**b**) symmetric and asymmetric dimers, (**c**) trigonal planar, T-shaped, and linear trimers.

Among the proton-bound noble gas clusters, the protonated noble gas monomers (NgH^+^) are the simplest. These species have a linear structure as shown in Figure 1a. The Cartesian coordinates for these structures are given in Section A of the Supplementary Material. Experimental studies have shown that the Ng-H^+^ internuclear distance increases from NeH^+^ to XeH^+^ [26,27,28,29]. As shown in Table 1, the experimental internuclear distances for NeH^+^, ArH^+^, KrH^+^, and XeH^+^are 0.9912, 1.2804, 1.4212, and 1.6028 Å, respectively. Such a trend is easily rationalized as the Ng’s size increases from Ne to Xe. Table 1 also shows the CCSD(T) equilibrium internuclear distance for NgH^+^. The predicted bond distance at this level of theory not only captures the trend, but also agrees well with the reported experimental values. Furthermore, our CCSD(T) internuclear distances have reproduced the reported calculations of Borocci et al. [51] Furthermore, our calculated internuclear distances for NeH^+^ and ArH^+^ agrees well with those reported by de Oca-Estévez et al., which were calculated at the CCSD(T)/CBS[56] and MRCI+Q/aug-cc-pV6Z levels of theory and basis [31].

As for the nature of the Ng-H^+^ bond, Borocci et al. have performed an extensive bonding analysis [51]. Their findings indicate that the nature of the Ng-H^+^ bond is dominantly covalent.

The dissociation of NgH^+^ can occur in two distinct pathways. The energetics for these pathways are compiled in Table 2. On the one hand the dissociation may lead to Ng and H^+^. The dissociation energy for this pathway is very high (>50 kcal/mol) [51,57,58,60]. The CCSD(T)/CBS//CCSD(T)/aug-cc-pVTZ dissociation energies are 53.03 kcal/mol for NeH^+^, 93.64 kcal/mol for ArH^+^, 104.79 kcal/mol for KrH^+^, and 120.01 kcal/mol for XeH^+^[60]. The increasing binding energy from NeH^+^ to XeH^+^ is attributed to the increasing polarizability of the Ng from Ne to Xe. We note that these values agree well with our previous dissociation energy calculations at the CCSD(T)/aug-cc-pVQZ//MP2/aug-cc-pVQZ level of theory and basis [58]. In our previous work, these dissociation energies are 53.43 kcal/mol for NeH^+^, 93.96 kcal/mol for ArH^+^, 105.94 kcal/mol for KrH^+^, and 121.43 kcal/mol for XeH^+^. The discrepancies between the present and previous results are attributed to the difference in reference geometry used and basis set size. The previous study used the MP2/aug-cc-pVQZ geometries, while the present study used the CCSD(T)/aug-cc-pVTZ geometries. Moreover, the present dissociation energy calculations are based on the CBS limit.

On the other hand, the dissociation could also lead to Ng^+^ and H. The dissociation energies at the CCSD(T)/CBS//CCSD(T)/aug-cc-pVTZ level for this pathway are 237.46 kcal/mol for NeH^+^, 144.95 kcal/mol for ArH^+^, 118.21 kcal/mol for KrH^+^, and 94.35 kcal/mol for XeH^+^. Notice that from NeH^+^to XeH^+^, the trends in dissociation energy between these two pathways are opposite. In the NgH^+^→ Ng + H^+^ pathway, the dissociation energy increases from NeH^+^ to XeH^+^, while in the NgH^+^→ Ng^+^ + H, the dissociation energy decreases from NeH^+^ to XeH^+^. We interpret these opposing trends as follows: In the NgH^+^→ Ng + H^+^ pathway, the polarizability of the Ng atom dictates the trend. However, in the NgH^+^→ Ng^+^ + H, the Ng atom’s ionization energy dictates the trend.

A comparison of the dissociation energy between these two pathways suggests that XeH^+^ might dissociate differently from the rest of the NgH^+^ complexes. As shown in Table 2, the NgH^+^→ Ng + H^+^ pathway is energetically favored for NeH^+^, ArH^+^, and KrH^+^, while the NgH^+^→ Ng^+^ + H pathway is more favored for XeH^+^. As a result, the XeH^+^ dissociates to Xe^+^ and H, while the rest of the NgH^+^ dissociates to Ng and H^+^. To understand this unusual behavior of XeH^+^, one needs to examine the ionization energies of H and Ng. The ionization energies in decreasing order are as follows: Ne (21.56 eV), Ar (15.76 eV), Kr (14.00 eV), H (13.59 eV), and Xe (12.13 eV) [91]. Notice that Ne, Ar, and Kr have a higher ionization energy than H, while the ionization energy of Xe is lower than that of H. Hence, in NeH^+^, ArH^+^, and KrH^+^, one would expect that the charge is more localized at the H atom, but in XeH^+^, the charge would be more localized on the Xe atom as the dissociation proceeds.

To investigate further, we examined the atomic charges as the internuclear distance is varied. Since the goal is to investigate the qualitative behavior of the atomic charges with respect to bond elongation, we have performed Mulliken population analysis [92] and examine the Mulliken charges at the MP2 level. For NeH^+^, ArH^+^, and KrH^+^, we found that at 10 Å, the H atom carries the positive charge, while the Ng atom has a zero Mulliken charge. Meanwhile for XeH^+^, the positive charge is more localized to the Xe atom when the internuclear distance is at 10 Å. In addition to the Mulliken population analysis, we have also performed natural population analysis (NPA) [93]. Both of these population analyses have predicted that at large internuclear distances (10 Å) the postive charge is localized at the H atom for NeH^+^, ArH^+^, KrH^+^, and XeH^+^. However, for XeH^+^, the positive charge tends to localize on the Xe atom. A comparison of the atomic charges from these population analyses can be found in Section B of the Supplementary Material.

Although it is worthwhile to examine the dipole moment function (DMF) with respect to internuclear distance, one should note that for charged systems the dipole moment function is not unique. In other words, it depends on the choice of coordinate’s origin [94].

We now examine the pure vibrational spectroscopy of NgH^+^. Table 1 also has the harmonic and anharmonic frequencies for NgH^+^. Examining the harmonic frequencies $\omega_{e}$, it is evident that from NeH^+^ to XeH^+^the harmonic frequencies $\omega_{e}$ decreases. Inclusion of anharmonicity leads to a lower oscillation frequency. Comparing our calculated anharmonic frequencies with the experimental frequencies, we note that these frequencies agree within 50 cm^−1^. There are several factors for such discrepancy. These include basis set truncation error, neglect of the rotational-vibrational interactions, as well as other factors in the experiment that were not considered in the present Hamiltonian. To assess the basis set effects, we have reconstructed the PES using the aug-cc-pVQZ and aug-cc-pV5Z basis sets for H, Ne, and Ar. The corresponding aug-cc-pVQZ-PP and aug-cc-pV5Z-PP basis sets were used for Kr and Xe. We found that the use of larger basis sets leads to a better agreement with the experimental values when compared with the use of aug-cc-pVTZ and aug-cc-pVTZ-PP. In particular, at the aug-cc-pV5Z basis set, the discrepancies between the calculated aharmonic and observed frequencies are 6, 10, 23, and 28 cm^−1^ for NeH^+^, ArH^+^, KrH^+^, and XeH^+^, respectively. The sensitivity of the anharmonic frequency with the basis set’s size can be found in Section C of the Supplementary Material.

**Table 1:** Comparison of experimental and calculated structural parameter and vibrational frequencies.

Species	Method	R (Å)	$\omega_{e}$ (cm^−1^)	$\nu_{\mathrm{anh}}$ (cm^−1^)	Ref.
NeH^+^	Expt.	0.9912	2904	2677 ^a^	[26]
	CCSD(T)/aug-cc-pVTZ	0.9923	2947	2710	This Work
	CCSD(T)/CBS[56]	0.9913	2904	2679	[31]
	MRCI+Q/aug-cc-pV6Z	0.9913	-	-	[31]
	Theory	0.9918	2897	2675	[95]
	Theory	0.9912	-	2678	[96]
	MRCI-DKH/ANO	0.9925	2897	2674	[97]
ArH^+^	Expt.	1.2804	2711	2588 ^a^	[27]
	CCSD(T)/aug-cc-pVTZ	1.2821	2730	2604	This Work
	CCSD(T)/CBS[56]	1.2810	2716	-	[31]
	MRCI+Q/aug-cc-pV6Z	1.2810	-	-	[31]
	CEPA	1.286	2723	2611 ^a^	[20]
	MR-AQCC/ANO	1.279	2739	2595 ^a^	[98]
	Theory	1.268	-	-	[99]
KrH^+^	Expt.	1.4212	2495	2398 ^a^	[28]
	CCSD(T)/aug-cc-pVTZ	1.4135	2540	2436	This Work
	CEPA	1.419	2896	2670 ^a^	[21]
XeH^+^	Expt.	1.6028	2270	2187 ^a^	[29]
	CCSD(T)/aug-cc-pVTZ	1.5980	2325	2237	This Work
	CEPA	1.611	2314	2231	[22]

^a^ Obtained from $\omega_{e}$ and $\omega_{e}x_{e}$, $v_{\mathrm{anh}}=\omega_{e}-2\omega_{e}x_{e}$.

**Table 2:** CCSD(T)/CBS//CCSD(T)/aug-cc-pVTZ ^a^ dissociation and isomerization energies in kcal/mol.

Process	Ne	Ar	Kr ^a^	Xe ^a^
Monomer				
NgH^+^→ Ng + H^+^	53.03 ^b^	93.64 ^b^	104.79 ^b^	120.01 ^b^
NgH^+^→ Ng^+^ + H	237.46	144.95	118.21	94.35
Dimer				
NgH^+^Ng → Ng + NgH^+^	15.78	15.42 ^c^	15.57 ^b^	14.27 ^b^
NgH^+^Ng → 2Ng + H^+^	68.81	109.06	120.36 ^b^	134.27 ^b^
Trimer				
Ng_3_H^+^ (T-shaped) → Ng + NgH^+^Ng	1.29	2.05 ^c^	2.38	2.75
Ng_3_H^+^ (Linear) → Ng + NgH^+^Ng	0.49	1.34 ^c^	1.84	2.45
Ng_3_H^+^ (T-shaped) → Ng_3_H^+^ (Linear)	0.80	0.70	0.54	0.30

^a^ For K_r_ and X_e_ atoms, aug-cc-pVXZ-PP (X = T, Q, 5) basis sets were used. ^b^ Adapted with permission from Ref. [60]. Copyright 2021 AIP Publishing. ^c^ Adapted with permission from Ref. [59]. Copyright 2020 American Chemical Society.

### 3.2. Proton-Bound Noble Gas Dimers

The proton-bound noble gas dimers are excellent systems to study the behavior of a proton sandwiched between two closed-shell species for several reasons. First, these systems are the simplest cationic proton-bound dimers (PBD) in terms of structure. Second, the species sandwiching the H^+^ are atomic species and have no internal structures—making it a good prototype species in studying PBDs. Third, the number of vibrational degrees of freedom (DOF) is relatively low (only four DOFs)—making the construction of a full-dimensional PES feasible.

Proton-bound noble gas dimers have a linear structure, which can be symmetric or asymmetric. In the symmetric proton-bound noble gas dimers (NgH^+^Ng), the proton is at the midpoint of the Ng-Ng distance, while in the asymmetric proton-bound noble gas dimers (NgH^+^Ng’), the proton is closer to the noble gas with a larger proton affinity (PA). Although these triatomic species both have a linear structure, it turns out that their vibrational structures are very distinct. Our previous detailed analysis have revealed that the symmetric NgH^+^Ng has a characteristic set of combination bands, while Fermi resonance is observed for a few selected asymmetric NgH^+^Ng’ species [58,60]. These spectral features will be elaborated in the ensuing discussions.

#### 3.2.1. Symmetric NgH^+^ Ng

Symmetric proton-bound noble gas dimers have a centrosymmetric linear structure. They belong to the $D_{\infty h}$ group. Table 3 shows the Ng-H^+^ internuclear distances for NgH^+^Ng. Similar to the trend in NgH^+^, these distances increases from NeH^+^Ne to XeH^+^Xe. The present CCSD(T) equilibrium are Ng-H^+^ distances are NeH^+^Ne (1.1395 Å), ArH^+^Ar (1.5058 Å), KrH^+^Kr (1.6519 Å), and XeH^+^Xe (1.8574 Å). Such trend is easily rationalized based on the atomic sizes of the noble gas atoms. A larger noble gas atom corresponds to a longer Ng-H^+^distance. Notice that the coordination of the second Ng to NgH^+^ leads to a significant elongation of the Ng-H^+^ bond—indicating an appreaciable interaction between the second Ng and NgH^+^complex. The Cartesian coordinates for these structures are given in Section D of the Supplementary Material.

In terms of energetics, the second Ng atom binds much weaker to the NgH^+^ complex when compared with the binding of an Ng with an isolated H^+^. Table 2 shows that the energy for the NgH^+^Ng → Ng + NgH^+^ process is between 14.27 and 15.78 kcal/mol. These energy requirements are much smaller than that of the NgH^+^→ Ng + H^+^ process which is in the 53.03–120.01 kcal/mol range (see Table 2). It is likely that the lesser charge of H^+^ in NgH^+^ leads to the weaker binding of the second Ng. Table 2 also compiles the energy needed to dissociate NgH^+^Ng to 2Ng and H^+^. All of these energies were calculated at the CCSD(T)/CBS//CCSD(T)/aug-cc-pVTZ level and basis set.

We now present the vibrational signatures of NgH^+^Ng. These species have four vibrational degrees of freedom. These are the symmetric Ng-H^+^ stretch $Q_{1}\left(\sigma_{g}^{+}\right)$, the doubly degenerate H^+^ bend $Q_{2}\left(\pi_{u}\right)$, and the asymmetric Ng-H^+^ stretch $Q_{3}\left(\sigma_{u}^{+}\right)$. Among these modes, $Q_{1}$ is infrared inactive, while both $Q_{2}$ and $Q_{3}$ are infrared active. Table 4 compiles both harmonic and anharmonic (1D, 2D, and 4D) frequencies for the fundamental bands of NgH^+^Ng. We will now systematically examine the effects of anharmonicity for these species. We begin by comparing harmonic and 1D anharmonic frequencies for each species. The 1D anharmonic calculation represents an uncoupled oscillator model, but accounts for the intrinsic anharmonicity of each modes only (i.e., anharmonic but no intermode coupling). As shown in Table 4, across all NgH^+^Ng species, $\nu_{1}$ bands are relatively harmonic. The harmonic and 1D anharmonic frequencies agree within 6 cm^−1^. As a result, any spectral shifts arising from higher-dimensional treatments for the $\nu_{1}$ band are mainly due to the intermode couplings. Meanwhile, a similar comparison for the $\nu_{2}$ band shows that the harmonic and 1D anharmonic frequencies agree within 28 cm^−1^. The most anharmonic band is the $\nu_{3}$ band, which corresponds to the asymmetric Ng-H^+^ stretch. A comparison of their harmonic and 1D anharmonic frequencies reveals a significant blue shift (>300 cm^−1^), when their intrinsic anharmonicities are considered. Such a significant shift implies that higher-order terms in the PES along the $Q_{3}$ mode are very crucial.

We now examine the effects of intermode coupling between $Q_{1}$ and $Q_{3}$ modes. In a more general sense, these two modes correspond to a proton stretch ($Q_{3}$) and proton donor–acceptor coordinate stretch ($Q_{1}$). The anharmonic frequencies when such coupling is included is provided in Table 4 under the 2D column header. When compared with the 1D anharmonic calculations, it is evident that the intermode coupling between $Q_{1}$ and $Q_{3}$ modes red shifts (>200 cm^−1^) the $\nu_{3}$ band. Such findings is consistent with our previous investigations on several proton-bound dimers (H_2_O)_2_H^+^, (MeOH)_2_H^+^, and (Me_2_O)_2_H^+^, as well as (NH_3_)_2_H^+^, (MeNH_2_)_2_H^+^, (Me_2_NH)_2_H^+^, and (Me_3_N)_2_H^+^ [94,100,101,102,103]. In all these species, the donor–acceptor coordinate plays an active role in modulating the frequency of the shared-proton stretch mode.

Lastly, a 4D anharmonic treatment is achieved when the doubly-degenerate H^+^ bend modes are allowed to couple with $Q_{1}$ and $Q_{3}$. The anharmonic frequencies when all of the vibrational modes are included are provided in Table 4 under the 4D column header. In this treatment, the proton’s 3D motion (two bends and one stretch) are fully considered. Since the $\nu_{3}$ band is the main intensity carrier, we will now analyze its spectral shifts when the bending modes were considered. Examining the shifts on the $\nu_{3}$ band as the dimensionality goes from 2D to 4D, it is evident that both $Q_{1}$ and $Q_{2}$ modes cooperatively red shifts the $\nu_{3}$ band across all NgH^+^Ng species considered in this work.

**Figure 2:**
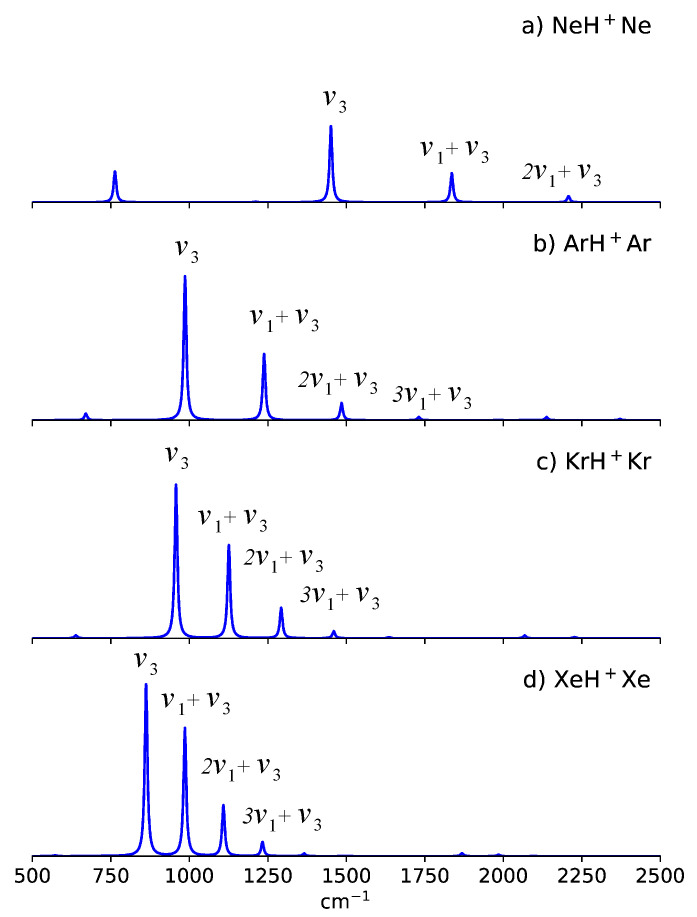
Anharmonic spectrum for (**a**) NeH^+^Ne, (**b**) ArH^+^Ar, (**c**) KrH^+^Kr, (**d**) XeH^+^ Xe. Subplot (**b**) was adapted with permission from Ref. [59]. Copyright 2020 American Chemical Society. Subplots (**c**,**d**) were adapted with permission from Ref. [60]. Copyright 2021 AIP Publishing.

In addition to these red shifts induced by the intermode coupling between $Q_{1}$ and $Q_{3}$ modes, an interesting spectral signature for NgH^+^Ng is also observed [57,58,59,60]. Figure 2 shows the 4D anharmonic spectrum for NgH^+^Ng. A strong progression of combination bands serves as a characteristic signature for these species. These combination bands involved one quantum of $\nu_{3}$ and multiple quanta of $\nu_{1}$, $n\nu_{1}+\nu_{3}$. These vibrational states all belong to the $\Sigma_{u}^{+}$ representation and is found to couple strongly with its neighboring states [58]. Simulations have suggested that these combination bands can efficiently borrow intensity from the $\nu_{3}$ band, which is the main intensity carrier [57,58]. Furthermore, the $\nu_{1}$ frequency decreases from NeH^+^Ne to XeH^+^Xe (mainly due to the mass-effects). As a result, the vibrational spacings between these bands decreases NeH^+^Ne to XeH^+^Xe, which in effect leads to a more efficient intensity redistribution as shown in Figure 2 [58]. A detailed analysis of the vibrational couplings is provided in ref. [58].

In our previous work, we have examined these symmetric NgH^+^Ng at the CCSD(T)/ aug-cc-pVQZ//MP2/aug-cc-pVQZ level of theory and basis set [58]. The $\nu_{3}$ peak positions between the previous and present work agree within 30 cm^−1^. As for the combination bands, the predicted peak positions agree within 35 cm^−1^. We note that although these shifts can be attributed to the basis set’s size, the predicted spectral features are similar. A bright $\nu_{3}$ band and a $n\nu_{1}+\nu_{3}$ progression band are the spectral signatures for NgH^+^Ng.

#### 3.2.2. Asymmetric NgH^+^ Ng’

We will now consider asymmetric proton-bound noble gas dimers, NgH^+^Ng’. Based on several studies, these triatomic NgH^+^Ng’ have a linear structure [35,37,45,51,60]. Compare with their symmetric counterparts (NgH^+^Ng), these species no longer have a center of inversion, because the Ng atoms sandwiching the H^+^ are now distinct (i.e., Ng ≠ Ng’). Table 5 shows the equilibrium distances for KrH^+^Ng and XeH^+^ Ng, (Ng = Ne, Ar, Kr, and Xe). Notice that the H^+^ prefers to be closer to the Ng with a higher proton affinity. The Cartesian coordinates for these structures are given in Section E of the Supplementary Material.

**Figure 3:**
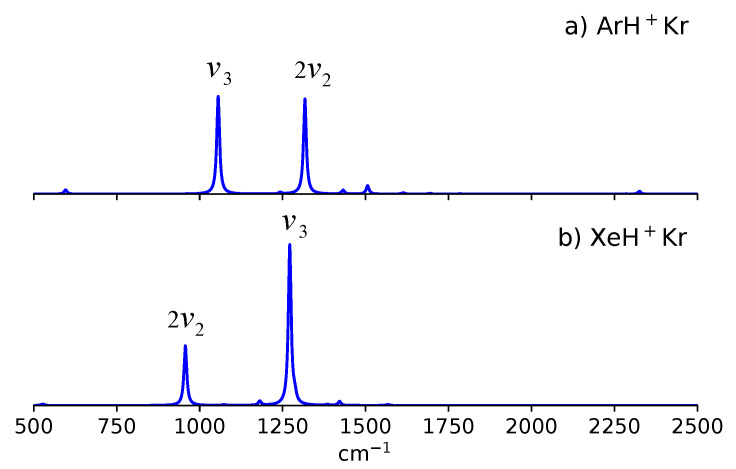
Fermi resonance bands between $2\nu_{2}$ and $\nu_{3}$ for (**a**) ArH^+^Kr and (**b**) XeH^+^Kr. Adapted with permission from Ref. [60]. Copyright 2021 AIP Publishing.

These asymmetric NgH^+^Ng’ species also have four vibrational degrees of freedom. These are the intermolecular Ng-Ng’ stretch $Q_{1}\left(\sigma^{+}\right)$, the doubly degenerate H^+^ bend $Q_{2}\left(\pi \right)$, and the shared-H^+^ stretch $Q_{3}\left(\sigma^{+}\right)$. Due to the absence of a center of inversion, all fundamentals are now infrared active. Hence, by symmetry considerations, both $n\nu_{1}+\nu_{3}\left(\sigma^{+}\right)$ combination bands as well as the $2\nu_{2}\left(\sigma^{+}\right)$ overtone band can now compete in borrowing intensity from the $\nu_{3}$ band. Our previous simulations have shown that ArH^+^Kr and KrH^+^Xe exhibits an appreciable Fermi resonance involving the $2\nu_{2}\left(\sigma^{+}\right)$ overtone and $\nu_{3}$ band [60]. The infrared spectra of these ArH^+^Kr and KrH^+^Xe are characterize by two intense bands in the 800–1400 cm^−1^ window, as shown in Figure 3.

In ArH^+^Kr, the Fermi resonance is manifested by peaks at 1056 cm^−1^ ($2\nu_{2}\left(\sigma^{+}\right)$) and 1317 cm^−1^ $\left(\nu_{3}\right)$. Meanwhile, in KrH^+^Xe, this Fermi resonance is exhibited by the bands at 957 cm^−1^ and 1271 cm^−1^ (Figure 2b). Recently, Tsuge and co-workers [37] have measured the vibrational spectra of KrH^+^Xe in *p*-H_2_ matrix. In their experiment, the $\nu_{3}$ band is located at 1284 cm^−1^, while a broad peak is also observed at 970 cm^−1^. These observed bands agree reasonably well with the predicted anharmonic bands.

As for the other asymmetric NgH^+^Ng’ species, previous works have indicated that the above Fermi resonance interaction is less favored [51,60]. The energy matching between the $2\nu_{2}\left(\sigma^{+}\right)$ overtone and $\nu_{3}$ bands for KrH^+^Ne, XeH^+^Ne, and XeH^+^Ar are not close enough to have a noticeable Fermi resonance [60]. A similar conclusion is drawn for NeH^+^Ng (Ng ≠ Ne or He) and ArH^+^Ng (Ng ≠ Ar) when their harmonic frequencies are examined [51].

### 3.3. Proton-Bound Noble Gas Trimers

The structure of proton-bound noble gas trimers provides crucial information on the first coordination sphere of the proton. Intuitively, there are three possible structures for Ng_3_H^+^. These are trigonal planar, T-shaped, and linear structures. These structures are illustrated in Figure 1c. In the trigonal planar structure, the Ng atoms are all equidistant to the H^+^, and their Ng-H^+^ distances are 120${}^{\circ }$ apart. Such structure belongs to the $D_{3h}$ symmetry group. Geometry minimization together with normal mode analysis at the CCSD(T) level reveal that these trigonal planar structures do not correspond to a minimum on the PES. Instead, these structures correspond to a second-order saddle point on the PES and possess two imaginary degenerate modes. These modes correspond to a proton motion towards one side of the equilateral triangle Ng framework. Such finding suggests that when Ng solvates the H^+^ in the gas phase, the first coordination sphere is comprised of two Ng atoms only. The succeeding Ng solvating the proton has to occupy the second coordination sphere, which makes the third Ng more distant to the H^+^ in comparison with the first two Ng atoms.

Further geometry minimization on the trigonal planar structure’s imaginary mode leads to a T-shaped Ng_3_H^+^ structure. In the T-shaped structure (Figure 1c), the distal Ng gas binds on top of the NgH^+^Ng structure and is located at the proton’s top. The two proximal Ng atoms are still equidistant from the H^+^. This T-shaped structure belongs to the $C_{2v}$ group. Comparing the structural parameters between NgH^+^Ng (Table 3) and T-shaped Ng_3_H^+^ (Table 6) shows that the Ng_proximal_-H^+^ distance does not change much when the third Ng binds to the NgH^+^Ng moiety.

In the linear Ng_3_H^+^ structure (Figure 1c), one Ng atom solvates the H^+^ on one side, while two well-separated Ng atoms solvate the H^+^ on the opposite side. The equilibrium position for the proton is closer towards the side with two Ng atoms. This linear structure belongs to the $D_{\infty h}$ symmetry group. The structural parameters for all three isomers of Ng_3_H^+^are compiled in Table 6. The Cartesian coordinates for these structures are given in Sections F and G of the Supplementary Material.

Among these two isomers for Ng_3_H^+^, the T-shaped isomer is more stable. However, the linear isomer is only within 0.80 kcal/mol higher in energy than the T-shaped isomer. Table 2 shows that the energetics for the T-shaped to liner isomerization decreases as the Ng gets heavier. The isomerization energies are Ne_3_H^+^ (0.80 kcal/mol), Ar_3_H^+^ (0.70 kcal/mol), Kr_3_H^+^ (0.54 kcal/mol), and Xe_3_H^+^ (0.30 kcal/mol).

The destruction of Ng_3_H^+^ to H^+^ and three Ng atoms can be examined from a stepwise Ng elimination point of view. The energies involved for these processes are compiled in Table 2. We first examine the energy required to remove one Ng from Ng_3_H^+^ (i.e., Ng_3_H^+^→ Ng + NgH^+^Ng). The energy required for this process is only between 1.29 and 2.75 kcal/mol for the T-shaped isomer. This energy goes down in the 0.49 to 2.45 kcal/mol range for the linear isomer. Meanwhile, the energy for the NgH^+^Ng → Ng + NgH^+^ process requires a much higher energy (14.27–15.78 kcal/mol). Lastly, elimination of Ng from the NgH^+^complex would need a much higher energy (53.03 to 120.01 kcal/mol). Based on the relative magnitudes of these energies, one may infer that a NgH^+^Ng core-ion is formed when Ng atoms solvate the H^+^. The Ng atoms of the core-ion binds much stronger than the additional Ng atoms. In the recent work of McDonald et al., calculations at the MP2/aug-cc-pVQZ level reveal that the ArH^+^Ar core-ion has five Ar atoms in its first solvation sphere [57].

Before discussing the anharmonic spectra for Ng_3_H^+^, we first examine the normal modes of its stable isomers. In addition to the core-ion modes, there are few low-frequency modes in both T-shaped and linear Ng_3_H^+^. For the T-shaped isomer, there are two low-frequency modes. These are the frustrated rotation of the NgH^+^Ng core-ion and the intermolecular stretch between the core-ion and distal Ng. Across all species, the harmonic frequencies for these modes are below 85 cm^−1^. Meanwhile for the linear isomer, there are three low-frequency modes in addition to the NgH^+^Ng core-ion modes. These are the doubly-degenerate frustrated rotation of the core-ion and the intermolecular stretch between the core-ion and Ng. The CCSD(T) harmonic frequencies for these modes are less than 60 cm^−1^.

In this work, we have ignored these low-frequency modes in the anharmonic spectra simulations for three main reasons. First, these frequencies are way too low. Including them in the anharmonic treatment would require us to solve a large number of eigenstates. Second, excited states for these low-frequency modes are often difficult to converge. Third, these low-frequency modes are outside the spectral region of interest. Therefore, we simulate the anharmonic spectrum using only the modes that corresponds to the core-ion.

**Figure 4:**
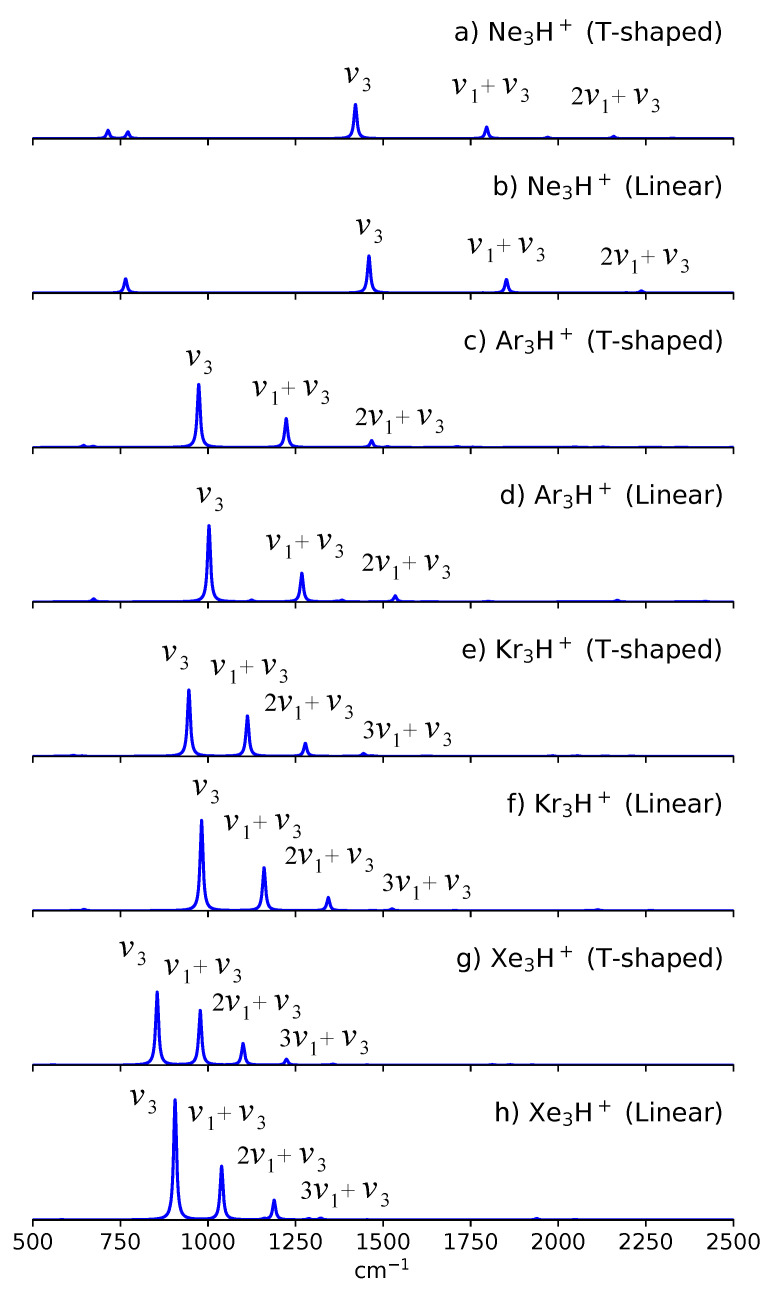
Anharmonic spectrum for (**a**) Ne_3_H^+^ (T-shaped), (**b**) Ne_3_H^+^ (Linear), (**c**) Ar_3_H^+^ (T-shaped), (**d**) Ar_3_H^+^ (Linear), (**e**) Kr_3_H^+^ (T-shaped), (**f**) Kr_3_H^+^ (Linear), (**g**) Xe_3_H^+^ (T-shaped), and (**h**) Xe_3_H^+^ (Linear). Subplots (**c**,**d**) are adapted with permission from Ref. [59]. Copyright 2020 American Chemical Society.

Figure 4 shows the 4D anharmonic spectra for both T-shaped and linear isomers of Ng_3_H^+^. In the T-shaped isomer, the core-ion’s symmetric Ng-H^+^ stretch $Q_{1}$ mode belongs to the $A_{1}$ representation, while the asymmetric Ng-H^+^ stretch $Q_{3}$ belongs to the $B_{2}$ representation. As a result, the $n\nu_{1}+\nu_{3}$ combination bands also belongs to the $B_{2}$ representation, which then makes the intensity borrowing between the $\nu_{3}$ and $n\nu_{1}+\nu_{3}$ bands still symmetry allowed. As shown in Figure 4, the spectra for the T-shaped isomers of Ng_3_ still exhibit the $n\nu_{1}+\nu_{3}$ combination bands. Notice that the $\nu_{3}$ and $n\nu_{1}+\nu_{3}$ bands are still very pronounced in the T-shaped Ng_3_H^+^. Furthermore, the trends in the strengths for these combination bands is similar to that in NgH^+^Ng. In other words, the $\nu_{3}$ band’s intensity increases from Ne_3_H^+^ to Xe_3_H^+^. Furthermore, the spacing of the combination bands decreases from Ne_3_H^+^ to Xe_3_H^+^, which then leads to a more efficient intensity redistribution.

We know turn our attention to the linear isomers of Ng_3_H^+^. In this case, both the core-ion’s symmetric Ng-H^+^ stretch $Q_{1}$ mode and asymmetric Ng-H^+^ stretch $Q_{3}$ belongs to the $\sigma^{+}$ representation. Hence, the $n\nu_{1}+\nu_{3}$ bands will also belong to the $\sigma^{+}$ representation. As a result, these combination bands are still allowed to borrow intensity from the bright $\nu_{3}$ band. As shown in Figure 4, these combination bands are also present in the linear isomers of Ng_3_H^+^.

In an actual experiment, both T-shaped and linear isomers are present in the sample. The observed spectrum may have peaks which can be attributed to each of these isomers. In the case of the Ar_3_H^+^, we found that although simulations for the T-shaped isomer can recover most of the experimental bands, simulations for both T-shaped and linear isomers are necessary to explain the observed bands at 1287, 1510, 1759, and 1993 cm^−1^ [57,59]. Furthermore, the peaks associated with the more stable T-shaped isomer dominate the experimental spectrum [59]. The observed strong band at 989 cm^−1^ agrees well with the predicted 973 cm^−1^ band, which then corresponds to the $\nu_{3}$ band of the T-shaped isomer. The experimental spectrum also has a very weak band around 1041 cm^−1^. Our previous simulation has attributed this band to the $\nu_{3}$ band of the linear isomer. We note that for Ar_3_H^+^, the $\nu_{3}$ band (973 cm^−1^) for the T-shaped isomer is lower than that of the linear isomer (1002 cm^−1^). A similar conclusion is drawn for the $\nu_{1}+\nu_{3}$ combination band. In the experimental spectrum the $\nu_{1}+\nu_{3}$ band for the T-shaped isomer is at 1237 cm^−1^, while for the linear isomer it is blue shifted to 1287 cm^−1^. These relative peak position were also captured in our simulations. In particular, the predicted $\nu_{1}+\nu_{3}$ peak positions are 1223 cm^−1^ for the T-shaped isomer and 1267 cm^−1^ for the linear isomer.

Table 7 compiles the predicted anharmonic frequencies for Ng_3_H^+^. We now compare the predicted $\nu_{3}$ and $n\nu_{1}+\nu_{3}$ peak positions for the linear and T-shaped isomers of the other Ng_3_H^+^ species. A comparison of the corresponding peaks between these isomers shows that the linear isomer’s peaks are also blue shifted relative to the T-shaped isomer’s peaks. In other words, the relative peak positions for the T-shaped and linear isomers are consistent across all trimer species considered in the present work. Unfortunately, experimental gas phase data for Ne_3_H^+^, Kr_3_H^+^, and Xe_3_H^+^ are not available at the moment. Furthermore, we note that although the present results have used a reasonable size for the basis set, the calculated peaks would be sensitive to the basis set’s size. However, the reported spectral features would be robust with respect to the basis set’s size. We hope that the presented results for Ng_3_H^+^ would motivate experimentalist to conduct more gas phase measurements in the future.

## 4. Conclusions

The characteristic spectral signatures of protonated noble gas clusters are examined from monomers to trimers. In the protonated monomers, the bright Ng-stretch band lies at the 2200–2700 cm^−1^ window. The frequency for this band red shifts as the noble gas gets heavier. Furthermore, the bonding energy for NgH^+^ is very strong. The addition of a second Ng atom to NgH^+^ leads to NgH^+^Ng, which is a prototypical system in the study of a more generic class of compounds know as the proton-bound dimers. Compare with the monomers, the symmetric NgH^+^Ng dimers have a unique spectral feature. In the mid-infrared region, a progression band is predicted for these complexes. This progression band involves one quantum of the asymmetric Ng-H^+^ stretch with multiple quanta of the symmetric Ng-H^+^ stretch $n\nu_{1}+\nu_{3}$. Present ab initio simulations shows that the strength of these progressions increases from NeH^+^Ne to XeH^+^Xe [58]. These progression bands are also very robust with respect to the addition of another Ng atom. In the Ng_3_H^+^, both T-shaped and linear isomers exhibit these progression bands. Meanwhile, for a few selected asymmetric NgH^+^Ng’, a Fermi resonance between the first H^+^ bend overtone and H^+^ stretch can be observed. Such anharmonic effect has not only been predicted, but also observed in ArH^+^Kr and XeH^+^Kr [37,60]. To date with, only the NgH^+^ and Ar_n_H^+^ (n = 3–7) have been measured in the gas phase. The remaining symmetric NgH^+^Ng as well as some of the asymmetric NgH^+^Ng’ has been extensively studied in several matrix environments. The authors hope that the present simulations will motivate the community to study these clusters more in the gas phase.

## Figures and Tables

**Table 3:** CCSD(T)/aug-cc-pVTZ ^a^ structural parameter and harmonic vibrational frequencies for Ng_2_H^+^.

Species	R (Å)	$\omega_{1}$ (cm^−1^)	$\omega_{2}$ (cm^−1^)	$\omega_{3}$ (cm^−1^)
NeH^+^Ne	1.1395	519	851	1618
ArH^+^Ar ^b^	1.5058	322	706	951
KrH^+^Kr ^c^	1.6519	208	678	943
XeH^+^Xe ^c^	1.8574	151	601	839

^a^ For K_r_ and X_e_ atoms, the aug-cc-pVTZ-PP basis set was used. ^b^ Adapted with permission from Ref. [59]. Copyright 2020 American Chemical Society. ^c^ Adapted with permission from Ref. [60]. Copyright 2021 AIP Publishing.

**Table 4:** Harmonic and anharmonic fundamental frequencies for NgH^+^ Ng.

Species	Band	Symmetry	Harmonic	1D	2D	4D
NeH^+^Ne	$\nu_{1}$	$\sigma_{g}^{+}$	519	513	476	460
	$\nu_{2}$	$\pi_{u}$	851	862	-	763
	$\nu_{3}$	$\sigma_{u}^{+}$	1618	2048	1600	1451
	$\nu_{1}+\nu_{3}$	$\sigma_{u}^{+}$			2004	1836
	$2\nu_{1}+\nu_{3}$	$\sigma_{u}^{+}$			2400	2201
ArH^+^Ar ^a^	$\nu_{1}$	$\sigma_{g}^{+}$	322	321	299	292
	$\nu_{2}$	$\pi_{u}$	706	734	-	670
	$\nu_{3}$	$\sigma_{u}^{+}$	951	1380	1083	986
	$\nu_{1}+\nu_{3}$	$\sigma_{u}^{+}$			1344	1237
	$2\nu_{1}+\nu_{3}$	$\sigma_{u}^{+}$			1601	1484
	$3\nu_{1}+\nu_{3}$	$\sigma_{u}^{+}$			1862	1730
KrH^+^Kr ^b^	$\nu_{1}$	$\sigma_{g}^{+}$	208	207	195	191
	$\nu_{2}$	$\pi_{u}$	678	694	-	638
	$\nu_{3}$	$\sigma_{u}^{+}$	943	1291	1040	957
	$\nu_{1}+\nu_{3}$	$\sigma_{u}^{+}$			1213	1126
	$2\nu_{1}+\nu_{3}$	$\sigma_{u}^{+}$			1386	1292
	$3\nu_{1}+\nu_{3}$	$\sigma_{u}^{+}$			1562	1460
XeH^+^Xe ^b^	$\nu_{1}$	$\sigma_{g}^{+}$	151	151	142	139
	$\nu_{2}$	$\pi_{u}$	601	618	-	572
	$\nu_{3}$	$\sigma_{u}^{+}$	839	1149	930	862
	$\nu_{1}+\nu_{3}$	$\sigma_{u}^{+}$			1057	986
	$2\nu_{1}+\nu_{3}$	$\sigma_{u}^{+}$			1184	1108
	$3\nu_{1}+\nu_{3}$	$\sigma_{u}^{+}$			1316	1233

^a^ Harmonic frequencies and 4D results are adapted with permission from Ref. [59]. Copyright 2020 American Chemical Society. ^b^ Harmonic frequencies and 4D results are adapted with permission from Ref. [60]. Copyright 2021 AIP Publishing.

**Table 5:** CCSD(T)/aug-cc-pVTZ ^a^ structural parameters for KrH^+^Ng and XeH^+^Ng ^b^.

NgH^+^Ng’	Ng-H (Å)	H-Ng’ (Å)
NeH^+^Kr	1.7765	1.4227
ArH^+^Kr	1.6895	1.5243
XeH^+^Kr	1.6984	1.8961
NeH^+^Xe	1.9980	1.6007
ArH^+^Xe	1.9497	1.6406
KrH^+^Xe	1.8961	1.6984

^a^ For K_r_ and X_e_ atoms, the aug-cc-pVTZ-PP basis set was used. ^b^ Data adapted with permission from Ref. [60]. Copyright 2021 AIP Publishing

**Table 6:** CCSD(T)/aug-cc-pVTZ ^a^ structural parameters for Ng_3_H^+^.

**Trigonal Planar**			
**Ng_3_H^+^**	**Ng-H (Å)**		
Ne_3_H^+^	1.3217		
Ar_3_H^+^ ^b^	1.7485		
Kr_3_H^+^	1.9086		
Xe_3_H^+^	2.1358		
**T-shaped Structure**			
**Ng_3_H^+^**	**Ng_proximal_-H (Å)**	**Ng_distal_-H (Å)**	
Ne_3_H^+^	1.1402	2.4950	
Ar_3_H^+^ ^b^	1.5057	3.2281	
Kr_3_H^+^	1.6516	3.4482	
Xe_3_H^+^	1.8564	3.7464	
**Linear Structure**	**Ng_a_-H^+^-Ng_b_ ⋯ Ng_c_**		
**Ng_3_H^+^**	**Ng_a_-H (Å)**	**Ng_b_-H (Å)**	**Ng_b_-Ng_c_ (Å)**
Ne_3_H^+^	1.1433	1.1350	2.7294
Ar_3_H^+^ ^b^	1.5327	1.4795	3.3822
Kr_3_H^+^	1.6950	1.6137	3.5294
Xe_3_H^+^	1.9396	1.7907	3.8152

^a^ For K_r_ and X_e_ atoms the aug-cc-pVTZ-PP basis set were used. ^b^ Adapted with permission from Ref. [59]. Copyright 2020 American Chemical Society.

**Table 7:** Anharmonic frequencies for the T-shaped and linear isomers of Ng_3_H^+^.

Species	Band	T-Shaped	Linear
Ne_3_H^+^	$\nu_{1}$	455	463
	$\nu_{2}$	715 (i.p.), 771 (o.p.)	765
	$\nu_{3}$	1421	1460
	$\nu_{1}+\nu_{3}$	1795	1852
	$2\nu_{1}+\nu_{3}$	2158	2237
Ar_3_H^+^ ^a^	$\nu_{1}$	290	292
	$\nu_{2}$	645 (i.p.), 671 (o.p.)	
	$\nu_{3}$	973	1002
	$\nu_{1}+\nu_{3}$	1223	1267
	$2\nu_{1}+\nu_{3}$	1466	1533
Kr_3_H^+^	$\nu_{1}$	190	189
	$\nu_{2}$	614 (i.p.), 639 (o.p.)	
	$\nu_{3}$	945	982
	$\nu_{1}+\nu_{3}$	1113	1160
	$2\nu_{1}+\nu_{3}$	1278	1343
	$3\nu_{1}+\nu_{3}$	1444	1525
Xe_3_H^+^	$\nu_{1}$	139	134
	$\nu_{2}$	557 (i.p.), 574 (o.p.)	582
	$\nu_{3}$	855	906
	$\nu_{1}+\nu_{3}$	978	1039
	$2\nu_{1}+\nu_{3}$	1100	1188
	$3\nu_{1}+\nu_{3}$	1224	1322

i.p. = in-plane, o.p. = out-of-plane. ^a^ Adapted with permission from Ref. [59]. Copyright 2020 American Chemical Society.

## Data Availability

Data are contained within the article and Supplementary Material.

## Supplementary Materials

The following supporting information can be downloaded at: https://www.mdpi.com/article/10.3390/molecules27103198/s1. Table S1: Cartesian coordinates (in Angstroms) for the CCSD(T) minimum structures for NgH^+^; Table S2: Comparison between the Mulliken charges and natural atomic charges for NgH^+^ when the internuclear distance is at 10 Å; Table S3: Sensitivity of the NgH^+^ anharmonic frequencies (cm^−1^) with basis set’s size; Table S4: Cartesian coordinates (in Angstroms) for the CCSD(T) minimum structures for NgH^+^Ng; Table S5: Cartesian coordinates (in Angstroms) for the CCSD(T) minimum structures for NgH^+^Ng’; Table S6: Cartesian coordinates (in Angstroms) for the CCSD(T) minimum structures for Ng_3_H^+^ T-shaped isomer; Table S7: Cartesian coordinates (in Angstroms) for the CCSD(T) minimum structures for Ng_3_H^+^ linear isomer; Table S8: Energy, zero-point energy, standard enthalpy, and standard free energies in Hartree.
