# Supplementary material for: Spectral Signatures of Protonated Noble Gas Clusters of Ne, Ar, Kr, and Xe: From Monomers to Trimers
Source: Molecules. 2022 May 17;27(10):3198. doi: 10.3390/molecules27103198 (PMC9143425; doi:10.3390/molecules27103198)
Supplement: Supplementary file 1 [file molecules-27-03198-s001.zip › molecules-1686889-supplementary.pdf]

Supplementary Material for

Spectral Signatures of Protonated Noble Gas Clusters of Ne, Ar, Kr, and Xe: From Monomers to Trimers

Jake A. Tan and Jer-Lai Kuo

Institute of Atomic and Molecular Sciences, Academia Sinica
No. 1 Roosevelt Rd., Sec. 4 Da-an District, Taipei City 10617, Taiwan (ROC)

Corresponding Authors:

Jake A. Tan Email: jaketan@gate.sinica.edu.tw

Jer-Lai Kuo Email: jlkuo@pub.iams.sinica.edu.tw

Table of Contents

A. Cartesian coordinates for the NgH^+ structures 3

B. Comparison of Mulliken Charges and Natural Atomic Charges 4

C. Sensitivity of the NgH^+ anharmonic frequencies with basis set's size 4

D. Cartesian coordinates for the NgH^+Ng structures 5

E. Cartesian coordinates for the NgH^+Ng' structures 6

F. Cartesian coordinates for the Ng_3H^+ T-shaped structures 7

G. Cartesian coordinates for the Ng_3H^+ linear structures 8

H. Compilation of CCSD(T)/aug-cc-pVTZ[a] electronic energy, zero-point energy, standard enthalpy, and standard free energies for protonated noble gas clusters 9

A. Cartesian coordinates for the NgH^+ structures

The aug-cc-pVTZ basis set was used for H, Ne, and Ar atoms, while the aug-cc-pVTZ-PP basis set was used for Kr and Xe atoms.

Table S1: Cartesian coordinates (in Angstroms) for the CCSD(T) minimum structures for NgH^+

**NeH^+**	X	Y	Z
H	0.000000	0.000000	0.000000
Ne	0.000000	0.000000	0.992291

**ArH^+**	X	Y	Z
H	0.000000	0.000000	0.000000
Ar	0.000000	0.000000	1.282051

**KrH^+**	X	Y	Z
H	0.000000	0.000000	0.000000
Kr	0.000000	0.000000	1.413533

**XeH^+**	X	Y	Z
H	0.000000	0.000000	0.000000
Xe	0.000000	0.000000	1.598010

B. Comparison of Mulliken Charges and Natural Atomic Charges

A comparison between the atomic charges (q) obtained from a Mulliken population analysis and natural population analysis is presented on the table below. The natural population analysis was conducted using the NBO 3.1 program, which is available in the Gaussian 16 Rev. A03 suite of programs. The calculations were conducted at the MP2 level of theory. The aug-cc-pVTZ basis set was used for H, Ne, and Ar atoms, while the aug-cc-pVTZ-PP basis set was used for Kr and Xe atoms.

Table S2: Comparison between the Mulliken charges and natural atomic charges for NgH^+ when the internuclear distance is at 10Å.

**Species**	**Mulliken Charges**		**Natural Atomic Charges**	
	q(Ng)	q(H)	q(Ng)	q(H)
**NeH^+**	0.00000	1.00000	-0.00019	1.00000
**ArH^+**	0.00000	1.00000	0.00000	1.00000
**KrH^+**	0.00000	1.00000	0.00000	1.00000
**XeH^+**	1.01159	-0.01159	0.99994	-0.01160

C. Sensitivity of the NgH^+ anharmonic frequencies with basis set's size

To assess the effect of the basis set's size on the anharmonic frequencies for NgH^+, we have built the one-dimensional anharmonic potential at the aug-cc-pVXZ (X=T, Q, and 5) basis sets. The corresponding aug-cc-pVXZ-PP (X=T, Q, and 5) were used for Kr and Xe atoms. To fully isolate the effect of the basis set's size the triple zeta minimum structures were used as the reference geometry, while the corresponding harmonic frequencies were used to define the Gauss-Hermite quadrature grids.

Table S3: Sensitivity of the NgH^+ anharmonic frequencies (cm^{-1}) with basis set's size.

**Basis Set[a]**	**NeH^+**	**ArH^+**	**KrH^+**	**XeH^+**
**aug-cc-pVTZ**	2710	2604	2436	2237
**aug-cc-pVQZ**	2692	2595	2417	2235
**aug-cc-pV5Z**	2683	2598	2421	2215

a) The Kr and Xe atoms, aug-cc-pVXZ-PP (X=T, Q, and 5) were used.

D. Cartesian coordinates for the NgH^+Ng structures

Table S4: Cartesian coordinates (in Angstroms) for the CCSD(T) minimum structures for NgH^+Ng

**NeH^+Ne**	X	Y	Z
H	0.000000	0.000000	0.000000
Ne	0.000000	0.000000	1.139526
Ne	0.000000	0.000000	-1.139526

**ArH^+Ar**	X	Y	Z
H	0.000000	0.000000	0.000000
Ar1	0.000000	0.000000	1.505819
Ar2	0.000000	0.000000	-1.505819

**KrH^+Kr**	X	Y	Z
H	0.000000	0.000000	0.000000
Kr	0.000000	0.000000	1.651906
Kr	0.000000	0.000000	-1.651906

**XeH^+Xe**	X	Y	Z
H	0.000000	0.000000	0.000000
Xe	0.000000	0.000000	1.857415
Xe	0.000000	0.000000	-1.857415

E. Cartesian coordinates for the NgH^+Ng' structures

Table S5: Cartesian coordinates (in Angstroms) for the CCSD(T) minimum structures for NgH^+Ng'

**NeH^+Kr**	X	Y	Z
H	0.000000	0.000000	-0.711734
Ne	0.000000	0.000000	-2.488247
Kr	0.000000	0.000000	0.71095

**ArH^+Kr**	X	Y	Z
H	0.000000	0.000000	-0.444815
Ar	0.000000	0.000000	-2.134319
Kr	0.000000	0.000000	1.079516

**XeH^+Kr**	X	Y	Z
H	0.000000	0.000000	-0.257742
Kr	0.000000	0.000000	-2.153823
Xe	0.000000	0.000000	1.440655

**NeH^+Xe**	X	Y	Z
H	0.000000	0.000000	-1.022404
Ne	0.000000	0.000000	-3.020352
Xe	0.000000	0.000000	0.578258

**ArH^+Xe**	X	Y	Z
H	0.000000	0.000000	-0.732868
Ar	0.000000	0.000000	-2.682581
Xe	0.000000	0.000000	0.907765

F. Cartesian coordinates for the Ng_3H^+ T-shaped structures

Table S6: Cartesian coordinates (in Angstroms) for the CCSD(T) minimum structures for Ng_3H^+ T-shaped isomer

Ne_3H^+	X	Y	Z
Ne	0.000000	0.000000	1.690174
H	0.000000	0.000000	-0.804845
Ne	0.000000	1.140219	-0.804845
Ne	0.000000	-1.140219	-0.804845

Ar_3H^+	X	Y	Z
Ar	0.000000	0.000000	2.171611
H	0.000000	0.000000	-1.056459
Ar	0.000000	1.505691	-1.056459
Ar	0.000000	-1.505691	-1.056459

Kr_3H^+	X	Y	Z
Kr	0.000000	0.000000	2.309320
H	0.000000	0.000000	-1.138843
Kr	0.000000	1.651551	-1.138843
Kr	0.000000	-1.651551	-1.138843

Xe_3H^+	X	Y	Z
Xe	0.000000	0.000000	2.505180
H	0.000000	0.000000	-1.241098
Xe	0.000000	1.856449	-1.241098
Xe	0.000000	-1.856449	-1.241098

G. Cartesian coordinates for the Ng_3H^+ linear structures

Table S7: Cartesian coordinates (in Angstroms) for the CCSD(T) minimum structures for Ng_3H^+ linear isomer

Ne_3H^+	X	Y	Z
Ne	0.000000	0.000000	-2.387227
H	0.000000	0.000000	-1.243891
Ne	0.000000	0.000000	-0.108869
Ne	0.000000	0.000000	2.620485

Ar_3H^+	X	Y	Z
Ar	0.000000	0.000000	-3.106371
H	0.000000	0.000000	-1.573695
Ar	0.000000	0.000000	-0.094181
Ar	0.000000	0.000000	3.287979

Kr_3H^+	X	Y	Z
Kr	0.000000	0.000000	-3.366750
H	0.000000	0.000000	-1.671785
Kr	0.000000	0.000000	-0.058093
Kr	0.000000	0.000000	3.471282

Xe_3H^+	X	Y	Z
Xe	0.000000	0.000000	-3.747465
H	0.000000	0.000000	-1.807847
Xe	0.000000	0.000000	-0.017128
Xe	0.000000	0.000000	3.798072

H. Compilation of CCSD(T)/aug-cc-pVTZ[a] electronic energy, zero-point energy, standard enthalpy, and standard free energies for protonated noble gas clusters

Table S8: Energy, zero-point energy, standard enthalpy, and standard free energies in Hartree

Monomers	E	ZPE	H (298.15 K, 1 atm)	G (298.15 K, 1 atm)
NeH^+	-128.897819	0.006715	-128.887800	-128.907720
ArH^+	-527.199804	0.006219	-527.190280	-527.211654
KrH^+	-462.630050	0.005787	-462.620958	-462.643561
XeH^+	-328.734262	0.005296	-328.725661	-328.749134

Dimers	E	ZPE	H (298.15 K, 1 atm)	G (298.15 K, 1 atm)
NeH^+Ne	-257.737161	0.008745	-257.724786	-257.749238
ArH^+Ar	-1054.27338	0.006119	-1054.263362	-1054.290458
KrH^+Kr	-925.115037	0.005710	-925.105189	-925.134599
XeH^+Xe	-657.297045	0.004994	-657.287724	-657.318839
NeH^+Kr	-591.447159	0.007232	-591.435170	-591.465004
ArH^+Kr	-989.695721	0.006375	-989.685159	-989.714465
XeH^+Kr	-791.208460	0.005941	-791.198128	-791.229577
NeH^+Xe	-457.549701	0.006458	-457.538166	-457.569680
ArH^+Xe	-855.792707	0.006382	-855.781727	-855.812754

Trimers (T-shaped)	E	ZPE	H (298.15 K, 1 atm)	G (298.15 K, 1 atm)
Ne_3H^+	-386.552085	0.008987	-386.537376	-386.570957
Ar_3H^+	-1581.325382	0.006287	-1581.312971	-1581.350523
Kr_3H^+	-1387.577217	0.005821	-1387.565033	-1387.605940
Xe_3H^+	-985.838738	0.005095	-985.827064	-985.870303

Trimers (Linear)	E	ZPE	H (298.15 K, 1 atm)	G (298.15 K, 1 atm)
Ne_3H^+	-386.550704	0.008947	-386.535484	-386.571855
Ar_3H^+	-1581.324280	0.006357	-1581.311316	-1581.350431
Kr_3H^+	-1387.576377	0.005966	-1387.563602	-1387.605251
Xe_3H^+	-985.838118	0.005350	-985.825752	-985.869327

a) The aug-cc-pVTZ-PP basis set was used for Kr and Xe atoms
